# Supplementary material for: Betulinic Acid Increases the Lifespan of Drosophila melanogaster via Sir2 and FoxO Activation
Source: Nutrients. 2024 Feb 1;16(3):441. doi: 10.3390/nu16030441 (PMC10856809; doi:10.3390/nu16030441)
Supplement: Supplementary file 1 [file nutrients-16-00441-s001.zip › nutrients-2821785-supplementary.pdf]

Figure S1

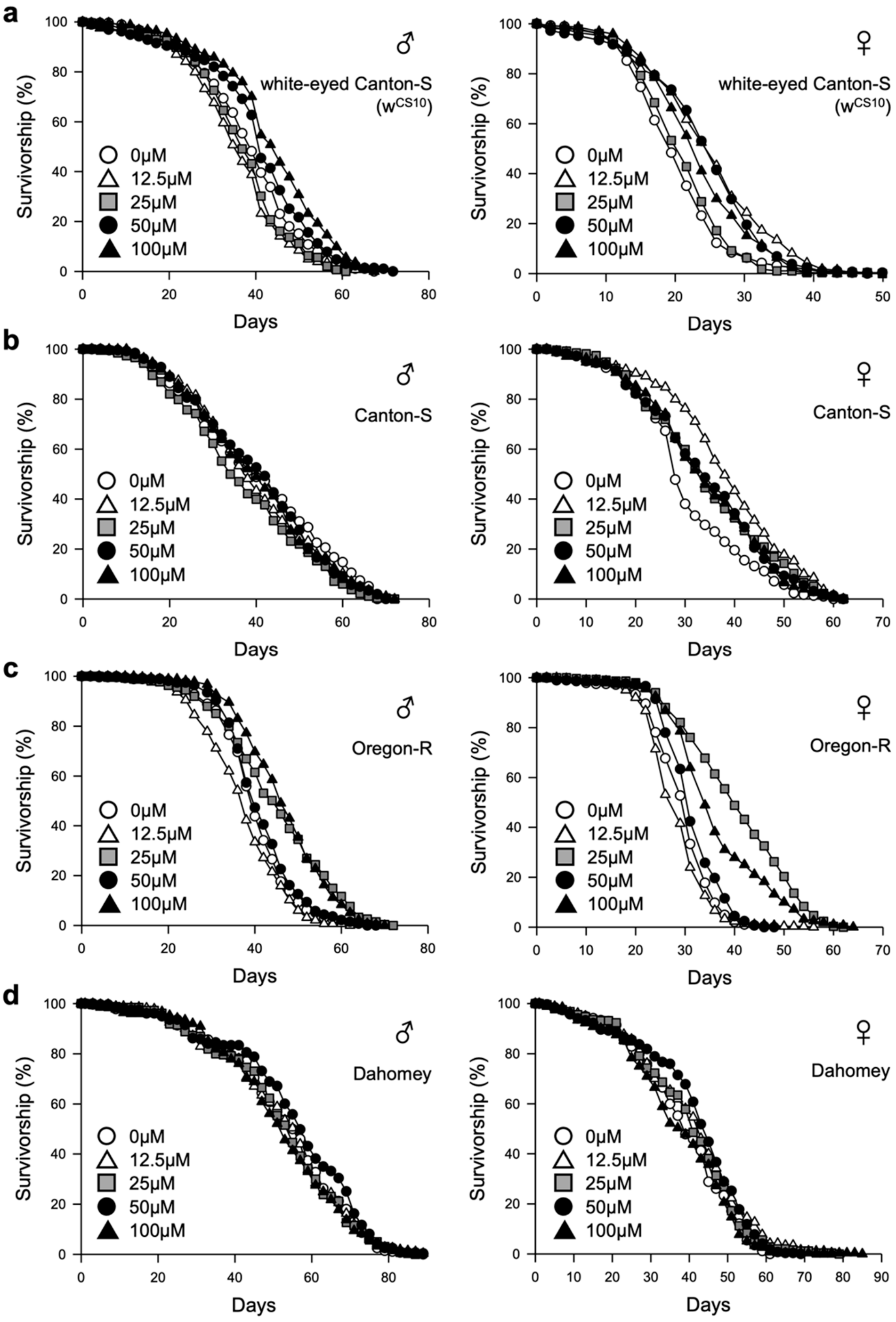

Figure S1. Effect of betulinic acid on lifespan in different strains. **(a-d)** Lifespan of white-eyed Canton-S (w^{CS10}) **(a)**, Canton-S **(b)**, Oregon-R **(c)**, and Dahomey **(d)**. Left panels show the lifespan of male. Right panels show the lifespan of female fruit flies.

Figure S2

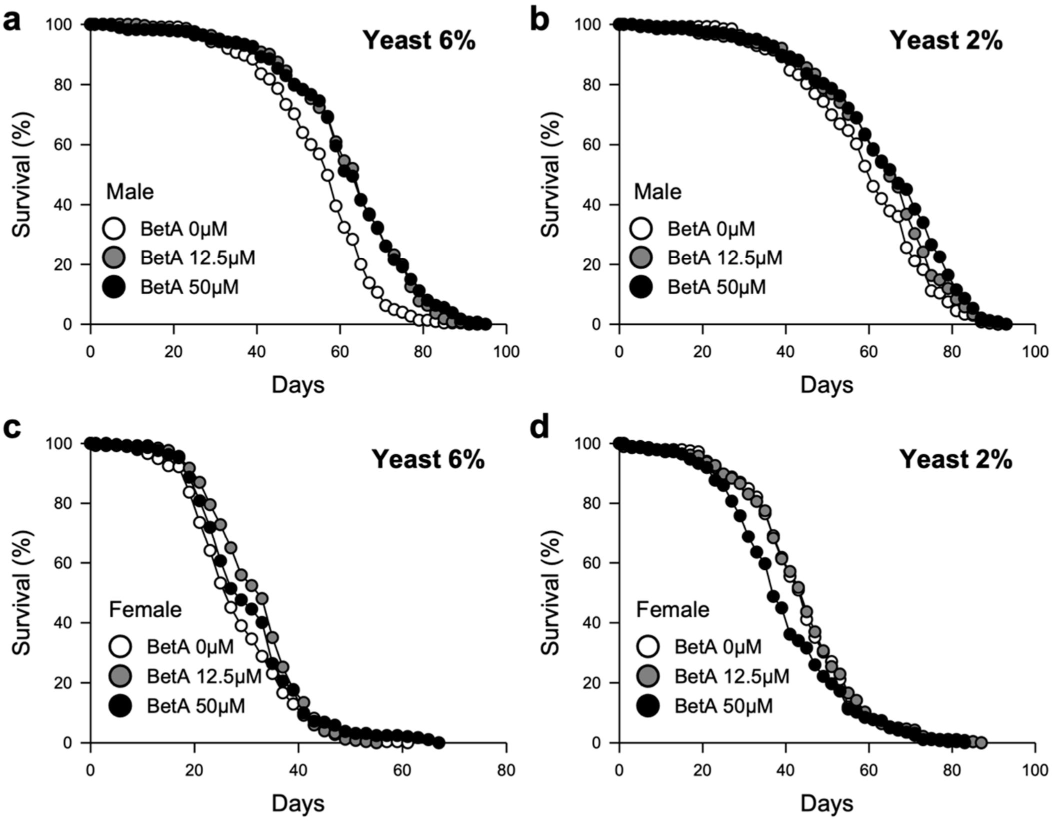

Figure S2. Relationship between the longevity effect of BetA and dietary restriction (DR) in fruit flies. **(a-b)** Survival curve of male flies fed BetA on 6% **(a)** or 2% Brewer's yeast diet **(b)**. **(c-d)** Survival curve of female flies fed BetA on 6% **(c)** or 2% Brewer's yeast diet **(d)**. The white circles indicate the lifespan of flies fed 0 μM BetA, the grey circles indicate the lifespan of flies fed 12.5 μM BetA, and the black circles indicate the lifespan of flies fed 50 μM BetA.

Table S1 Composition of fly husbandry food

Food	Composition
Cornmeal-sugar-yeast (CSY) media	5.2% cornmeal
	11% sugar
	2.6% instant yeast
	0.5% propionic acid
	0.04% methyl 4 hydroxybenzoate (Sigma Aldrich, MO, USA)
Sugar-yeast (SY) media	10% sugar
	10% yeast
	0.8% agar
	0.5% propionic acid
	0.04% methyl 4 hydroxybenzoate (Sigma Aldrich, MO, USA)
Dietary restriction experiment	For DR condition, 2% Brewer's yeast<br>For FD condition, 6% Brewer's yeast
	10% sugar
	0.8% agar
	0.5% propionic acid
	0.04% methyl 4 hydroxybenzoate (Sigma Aldrich, MO, USA)

Table S2 Sequences of primers for PCR

Primer	Forward (5'-3')	Reverse (5'-3')
rp49	ATC GGT TAC GGA TCG AAC AA	GAC AAT CTC CTT GCG CTT CT
catalase (*cat*)	TAC GAG CAG GCC AAG AAG TT	ACC TTG TAC GGG CAG TTC AC
sod1	GTT CGG TGA CAA CAC CAA TG	GGA GTC GGT GAT GTT GAC CT
sod2	TCT GAA GAA GGC CAT CGA GT	GCA GAT AGT AGG CGT GCT CC
gs	TGG GAC CAG CAA GTA AAA CC	TCG CGA ATG TAG AAC TCG TG
thioredoxin (*trx*)	AAT GTG CTG GAG CTC TTC GT	TTG TTG TCG TTG TCG CTT TC
dilp1	AAT GGC AAT GGT CAC GCC GAC TGG	GCT GTT GCC CAG CAA GCT TTC ACG
dilp2	ACG AGG TGC TGA GTA TGG TGT GCG	CAC TTC GCA GCG GTT CCG ATA TCG
dilp3	GTC CAG GCC ACC ATG AAG TTG TGC	CTT TCC AGC AGG GAA CGG TCT TCG
dilp4	TGG ATT TAC ACG CCG TGT CAG GCG	ACA CCC TTC TCC GTA TCC GCA TGG
dilp5	TGT TCG CCA AAC GAG GCA CCT TGG	CAC GAT TTG CGG CAA CAG GAG TCG
dilp6	TGC TAG TCC TGG CCA CCT TGT TCG	GGA AAT ACA TCG CCA AGG GCC ACC
dilp7	GAG CTG TAC TCC TGT TCG TCC TGC	TCC AAG CCT CAT CAT TGC CCG TCC
sir2	CAC GAC CGT TCT ACA AGT TT	GCA GCT CCT CCT CAG TAA C
thor	GAA GGT TGT CAT CTC GGA TCC	ATG AAA GCC CGC TCG TAG
impl2	GCC GAT ACC TTC GTG TAT CC	TTT CCG TCG TCA ATC CAA TAG
Inr	TAC TCG GAG CAT TGG AGG CAT	AAC AGT GGC GGA TTC GGT T
l(2)efl	AGG GAC GAT GTG ACC GTG TC	CGA AGC AGA CGC GTT TAT CC

Table S3 Effect of BetA on lifespan in fruit flies

Sex	Betulinic Acid (μM)	n	Mean-lifespan	Change (vs. 0 μM)	Median-lifespan	Maximum-lifespan	χ^2 (vs. 0 μM)	*p*-value (vs. 0 μM)	Mortality	R^2
Male	0	299	44.84 ± 0.63		45	51			y = 0.1811x - 6.7948	0.8908
	10	301	49.96 ± 0.65	11%	51	57	34.1957	< 0.0001*	y = 0.1944x - 7.4026	0.9153
	25	310	47.84 ± 0.71	7%	49	57	15.691	< 0.0001*	y = 0.2045x - 7.352	0.9051
	50	296	50.82 ± 0.68	13%	53	59	51.0212	< 0.0001*	y = 0.1156x - 5.7423	0.5614
	100	300	47.73 ± 0.70	6%	51	55	15.3152	< 0.0001*	y = 0.182x - 6.8224	0.8616
Female	0	285	42.31 ± 0.68		43	49			y = 0.2142x - 6.8755	0.8957
	10	278	44.14 ± 0.75	4%	47	55	6.6335	0.0100*	y = 0.1905x - 6.4423	0.9016
	25	279	44.11 ± 0.77	4%	45	53	7.704	0.0055*	y = 0.1959x - 6.4437	0.9242
	50	269	44.73 ± 0.79	6%	47	55	12.0737	0.0005*	y = 0.1741x - 6.4269	0.9452
	100	287	42.58 ± 0.72	1%	43	51	0.6566	0.4177	y = 0.2028x - 6.4784	0.9404

Table S4 Effect of BetA on lifespan in fruit flies on DR condition

Sex	Group		n	Mean-lifespan	Change (vs. 0 μM)	Median-lifespan	Maximum-lifespan	χ^2 (vs. 0 μM)	*p*-value (vs. 0 μM)
Male	Yeast 2%	0 μM	269	59.57 ± 0.94		61	71		
		12.5 μM	277	62.48 ± 0.98	5%	65	73	6.1229	0.0133*
		50 μM	244	63.94 ± 1.09	7%	67	77	16.2592	< 0.0001*
	Yeast 6%	0 μM	225	55.41 ± 0.89		57	65		
		12.5 μM	271	62.14 ± 0.91	12%	65	73	41.9287	< 0.0001*
		50 μM	287	62.14 ± 0.95	12%	63	73	43.946	< 0.0001*
Female	Yeast 2%	0 μM	285	47.72 ± 0.80		49	57		
		12.5 μM	284	47.96 ± 0.84	1%	49	57	0.2482	0.6183
		50 μM	285	43.65 ± 0.85	-9%	41	53	7.5816	0.0059*
	Yeast 6%	0 μM	295	34.55 ± 0.57		33	41		
		12.5 μM	254	37.96 ± 0.55	10%	39	45	9.26	0.0023*
		50 μM	292	36.71 ± 0.61	6%	35	43	6.0009	0.0143*

Table S5 Effect of BetA on lifespan in mutant fruit flies

Sex	Strain	Group	n	Mean-lifespan	Change (vs. 0 μM)	Median-lifespan	Maximum-lifespan	χ^2 (vs. 0 μM)	*p*-value (vs. 0 μM)
Male	w^{1118}	0 μM	266	68.37 ± 0.78		69	77		
		50 μM	290	72.28 ± 0.79	6%	75	79	16.9553	< 0.0001*
	$sir2^{4.5/5.26}$	0 μM	259	66.81 ± 0.81		69	75		
		50 μM	282	67.29 ± 0.61	1%	69	75	1.2807	0.2578
	yw	0 μM	299	30.83 ± 0.82		30	40		
		50 μM	265	39.54 ± 0.92	28%	42	50	47.8442	< 0.0001*
	$foxo^{25/21}$	0 μM	323	52.24 ± 0.97		56	68		
		50 μM	286	54.95 ± 0.96	5%	60	68	0.5619	0.4535
Female	w^{1118}	0 μM	297	56.26 ± 0.91		53	69		
		50 μM	310	64.32 ± 0.94	14%	67	77	31.3537	< 0.0001*
	$sir2^{4.5/5.26}$	0 μM	279	55.92 ± 0.78		59	63		
		50 μM	272	57.51 ± 0.68	3%	59	65	0.1711	0.6791
	yw	0 μM	295	50.47 ± 0.90		55	63		
		50 μM	271	54.17 ± 0.90	7%	59	65	12.4621	0.0004*
	$foxo^{25/21}$	0 μM	277	58.39 ± 0.88		61	69		
		50 μM	250	55.65 ± 1.14	-5%	61	67	0.0037	0.9513
